# Restless Legs Syndrome Associated With Fexofenadine Use: A Case Report

**DOI:** 10.1002/ccr3.71962

**Published:** 2026-01-28

**Authors:** Abdulrahman M. Albeshry

**Affiliations:** ^1^ Department of Family and Community Medicine, Faculty of Medicine University of Jeddah Jeddah Saudi Arabia

**Keywords:** case report, fexofenadine, restless legs syndrome, second‐generation antihistamines

## Abstract

Restless legs syndrome (RLS) is a neurological sensorimotor disorder characterized by an irresistible urge to move the legs, typically worsening at night and leading to sleep disturbance. While first‐generation antihistamines are recognized as common pharmacological triggers, second‐generation nonsedating antihistamines are rarely implicated. We report the case of a 35‐year‐old man with allergic rhinitis who developed new‐onset RLS after 1 month of treatment with fexofenadine 180 mg daily. His symptoms included an uncomfortable urge to move his legs, particularly in the evening and at night, which interfered significantly with sleep quality. Clinical examination and laboratory studies, including renal function and iron indices, were unremarkable. Despite several medical consultations, the symptoms persisted until fexofenadine was discontinued. Remarkably, his symptoms resolved completely within days of withdrawal without pharmacologic intervention and did not recur on follow‐up. This case highlights a rare but clinically relevant association between fexofenadine and RLS. Although fexofenadine is classified as a peripherally selective, nonsedating antihistamine, this report underscores that even second‐generation agents may contribute to RLS in susceptible individuals. Recognition of this potential adverse effect is important, as prompt discontinuation of the offending medication alone can result in rapid and complete symptom resolution. Clinicians should therefore maintain vigilance when evaluating patients with new‐onset RLS and carefully review medication histories, including use of second‐generation antihistamines such as fexofenadine.

## Key Clinical Message

Fexofenadine, though considered a nonsedating second‐generation antihistamine, can rarely trigger restless legs syndrome (RLS). Clinicians should consider drug‐induced RLS in patients presenting with new‐onset nocturnal restlessness, as symptoms may completely resolve after discontinuation of fexofenadine.

## Graphical abstract

Flowchart of possible fexofenadine‐induced RLS pathophysiology.

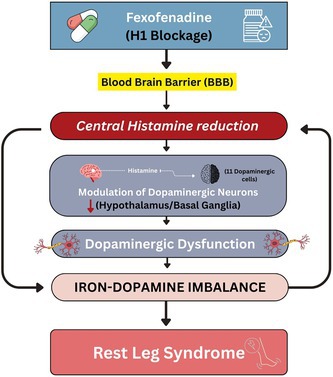

## 1 Introduction

Restless legs syndrome (RLS), also known as Willis‐Ekbom disease, is a common neurological sensorimotor disorder characterized by an irresistible urge to move the legs, typically accompanied by uncomfortable or unpleasant sensations [1]. The condition affects approximately 3%–7% of the adult population globally, with higher prevalence rates in European regions and women being affected twice as frequently as men [2]. While RLS can occur as a primary idiopathic condition with strong genetic components, secondary forms can result from various medical conditions, medications, and physiological states.

Fexofenadine is a second‐generation, nonsedating H1 antihistamine that selectively antagonizes peripheral histamine receptors without significant central nervous system penetration [3]. As the active metabolite of terfenadine, fexofenadine exhibits high specificity for H1 receptors with minimal anticholinergic, antidopaminergic, or sedative effects. Despite its classification as nonsedating due to limited blood–brain barrier penetration, emerging rare case reports have documented fexofenadine‐induced RLS [4].

The first documented case of fexofenadine‐induced RLS involved a 30‐year‐old woman who developed symptoms after 3 months of fexofenadine/pseudoephedrine treatment. The temporal relationship between medication initiation and symptom onset, combined with rapid symptom resolution following discontinuation, strongly suggested a causal relationship [4]. While traditional first‐generation antihistamines are well‐established RLS triggers due to high central H1 receptor occupancy, the mechanism underlying RLS induced by second‐generation antihistamines remains unclear [5].

Antihistamines have been identified among the top medication classes significantly associated with elevated RLS risk, with diphenhydramine demonstrating the strongest evidence base. The pathophysiology may involve complex interactions between histaminergic and dopaminergic systems, as histamine serves as both a neurotransmitter and neuromodulator in brain regions critical for RLS development [6].

This case challenges traditional assumptions about second‐generation antihistamine safety in movement disorder‐susceptible patients. It underscores the importance of comprehensive medication history assessment in patients presenting with new‐onset RLS symptoms.

## 2 Case History/Examination

A 35‐year‐old male with a past medical history significant only for allergic rhinitis presented with a 1‐month history of disturbing restlessness in both legs. The symptoms were described as an irresistible urge to move, occurring in the evening and worsening at night, interfering with sleep. He denied any history of neurological disorders, renal disease, iron deficiency, or use of other medications.

On physical examination, his vital signs were within normal limits (blood pressure 110/70 mmHg, heart rate 72 beats/min, respiratory rate 14 breaths/min, and body temperature 36.7°C). Examination of the cardiovascular, respiratory, and gastrointestinal systems did not reveal any abnormal findings. There was no leg edema, rash, or other systemic abnormalities. Neurological examination was unremarkable, with preserved tendon reflexes and no sensory or motor deficits.

## 3 Differential Diagnosis, Investigations and Treatment

Laboratory investigations showed a white blood cell count of 5100/μL with 56% neutrophils, hemoglobin 13.8 g/dL with mean corpuscular volume of 94 fL, and a platelet count of 220 × 10^4^/μL. Serum biochemistry, including renal function tests, was within normal limits (blood urea nitrogen 13 mg/dL, creatinine 0.8 mg/dL). C‐reactive protein was 0.02 mg/dL, and ferritin was 65 ng/mL, excluding iron deficiency.

The patient reported that he had been taking fexofenadine 180 mg once daily for allergic rhinitis during the same period his symptoms developed, suspecting a drug‐induced etiology.

## 4 Conclusion and Results

Fexofenadine was discontinued. Remarkably, within a few days, the patient experienced complete resolution of symptoms. Unlike other reported cases where pramipexole was tried as symptomatic therapy, our patient required no additional pharmacologic intervention.

He was followed for several weeks, during which he remained symptom‐free, with no recurrence after withdrawal of fexofenadine. This temporal relationship strongly suggested a diagnosis of restless legs syndrome induced by fexofenadine.

## 5 Discussion

The present case, in conjunction with the previously published report, provides further evidence that fexofenadine, a second‐generation nonsedating antihistamine, may precipitate restless legs syndrome (RLS).

While RLS is classically associated with iron deficiency, renal disease, pregnancy, and medications such as antidepressants or dopamine antagonists, antihistamines, especially first‐generation sedating agents, are among the most frequently implicated drug classes [7]. The occurrence of RLS with fexofenadine, despite its peripheral selectivity and low central H1 receptor occupancy, raises important questions about underlying mechanisms.

**FIGURE 1:**
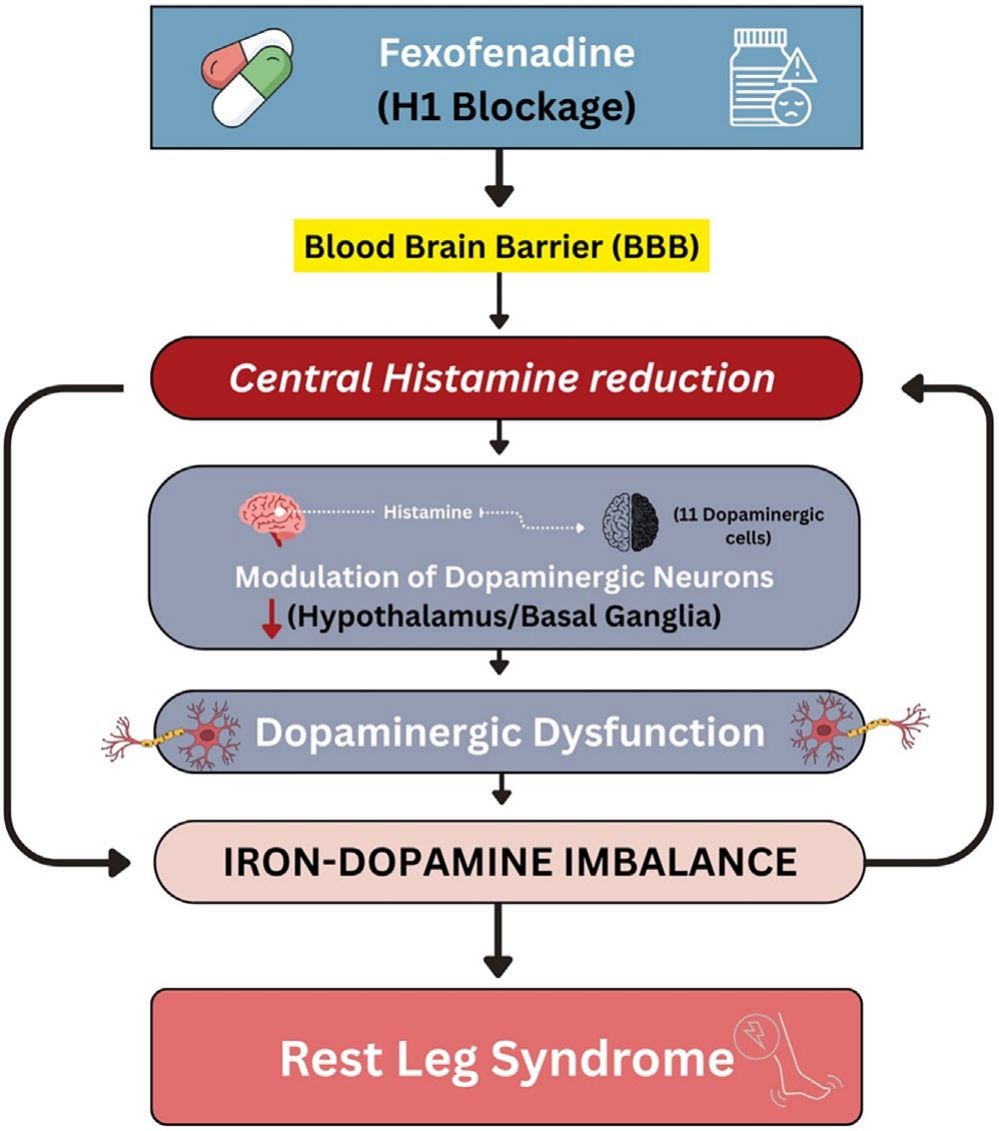
Proposed pathophysiological mechanism of fexofenadine‐induce restless leg syndrome (RLS). The diagram illustrate how fexofenadine crosses blood brain barrier, leading to central histamine reduction, modulation of dopaminergic neurons in the hypothalamus and basal ganglia, and subsequent iron dopamine imbalance resulting in RLS symptoms.

One possible explanation lies in histaminergic–dopaminergic interactions (as shown in Figure 1). Histamine serves not only as a mediator of wakefulness but also as a neuromodulator within basal ganglia pathways that regulate movement. In animal models, reduced histaminergic tone alters dopaminergic firing patterns, particularly within the substantia nigra and striatum. Fexofenadine's antagonism of H1 receptors, even at low central concentrations, may disrupt this delicate balance. In predisposed individuals, this could unmask latent dopaminergic dysfunction, leading to the sensorimotor manifestations of RLS [8].

Another plausible mechanism involves brain iron homeostasis. Iron deficiency, even when not reflected in systemic ferritin levels, has been demonstrated in the substantia nigra and thalamus of RLS patients. Iron is an essential cofactor for tyrosine hydroxylase, the rate‐limiting enzyme in dopamine synthesis [9]. Subclinical alterations in iron metabolism may render dopaminergic pathways more sensitive to pharmacologic perturbations, including reduced histaminergic input. Thus, fexofenadine may act as a “second hit” in susceptible individuals with marginal iron reserves.

Additional neurotransmitter systems may also contribute. Histamine interacts with adenosine and glutamatergic signaling, both of which are dysregulated in RLS. Blockade of central H1 receptors could reduce inhibitory adenosine tone and enhance excitatory glutamatergic activity, promoting hyperexcitability of spinal circuits and reinforcing the urge to move [8, 10]. This hypothesis is supported by emerging evidence that histamine H3 receptor antagonists, which increase histaminergic neurotransmission, can improve RLS symptoms, suggesting that impaired histamine signaling is directly relevant to disease mechanisms.

The comparison between our case and the earlier female patient further supports these mechanistic considerations. In that report, prolonged exposure to fexofenadine/pseudoephedrine preceded symptom onset, whereas in our patient, standard‐dose fexofenadine monotherapy induced RLS within 1 month. The shared outcome of symptom remission after drug discontinuation strongly implicates fexofenadine, regardless of pseudoephedrine coadministration [4]. These cases highlight that individual susceptibility—whether through blood–brain barrier permeability, genetic variation in drug metabolism, or subclinical iron–dopamine imbalance—may determine who develops this adverse effect.

Clinically, these findings underscore the importance of maintaining a broad differential when evaluating patients with new‐onset RLS. Even second‐generation antihistamines, typically regarded as safe alternatives, should not be excluded. Medication withdrawal should be considered early, as resolution may occur without the need for additional dopaminergic therapy.

## 6 Conclusion

Fexofenadine, despite its reputation as a nonsedating and peripherally selective antihistamine, can rarely induce RLS. Mechanistically, this may occur through disruption of histaminergic modulation of dopaminergic pathways, exacerbation of subclinical brain iron deficiency, and downstream effects on adenosine and glutamatergic systems. The accumulating case evidence—now in both male and female patients, with and without pseudoephedrine—emphasizes the need for clinicians to recognize this potential adverse reaction. Careful medication history‐taking and prompt discontinuation of the offending agent remain the cornerstone of management.

## Author Contributions

**Abdulrahman M. Albeshry:** conceptualization, data curation, formal analysis, funding acquisition, investigation, methodology, project administration, resources, validation, visualization, writing – original draft, writing – review and editing.

## Funding

The author has nothing to report.

## Consent

Written informed consent from the patient was obtained according to journal guidelines.

## Conflicts of Interest

The author declares no conflicts of interest.

## Data Availability

Data sharing is not applicable to this article as no datasets were generated or analyzed during the preparation of this case report. All information supporting the findings is contained within the manuscript.

## References

[1] A. Mansur , P. R. Castillo , F. R. Cabrero , and S. R. A. Bokhari , “Restless Legs Syndrome,” in The Curated Reference Collection in Neuroscience and Biobehavioral Psychology (University of Oxford, 2023), 131–135, https://www.ncbi.nlm.nih.gov/books/NBK430878/.

[2] B. Nanayakkara , J. Di Michiel , and B. J. Yee , “Restless Legs Syndrome,” Australian Journal of General Practice 52, no. 9 (2023): 615–621. 37666782 10.31128/AJGP-02-23-6722

[3] M. Panzarea , M. C. Astuto , and I. Cattaneo , “Fexofenadine,” in Encyclopedia of Toxicology Volume 1–9, vol. 4, Fourth ed. (StatPearls, 2024), V4‐713–V4‐719, https://www.ncbi.nlm.nih.gov/books/NBK556104/.

[4] H. Nishioka and Y. Kanzawa , “Restless Legs Syndrome Induced by Fexofenadine/Pseudoephedrine,” Journal of General and Family Medicine 21, no. 6 (2020): 256–257, 10.1002/jgf2.338. 33304720 PMC7689230

[5] R. P. Allen , A. S. Walters , J. Montplaisir , et al., “Restless Legs Syndrome Prevalence and Impact: REST General Population Study,” Archives of Internal Medicine 165, no. 11 (2005): 1286–1292, 10.1001/archinte.165.11.1286. 15956009

[6] S. Wei , X. Song , R. Chen , S. Chen , and B. Lu , “Exploring the Top 50 Drugs Associated With Restless Legs Syndrome Based on the FDA Data From 2004 to 2024,” Nature and Science of Sleep 17 (2025): 929, 10.2147/NSS.S513538. PMC12087588 40391202

[7] D. L. Bliwise , R. H. Zhang , and N. G. Kutner , “Medications Associated With Restless Legs Syndrome: A Case Control Study in the US Renal Data System (USRDS),” Sleep Medicine 15, no. 10 (2014): 1241, https://pmc.ncbi.nlm.nih.gov/articles/PMC4172448/. 25156752 10.1016/j.sleep.2014.05.011 PMC4172448

[8] K. Yanai and M. Tashiro , “The Physiological and Pathophysiological Roles of Neuronal Histamine: An Insight From Human Positron Emission Tomography Studies,” Pharmacology & Therapeutics 113, no. 1 (2007): 1–15, 10.1016/j.pharmthera.2006.06.008. 16890992

[9] S. Y. Koh , M. S. Kim , S. M. Lee , J. M. Hong , and J. H. Yoon , “Impaired Vascular Endothelial Function in Patients With Restless Legs Syndrome: A New Aspect of the Vascular Pathophysiology,” Journal of the Neurological Sciences 359, no. 1–2 (2015): 207–210, 10.1016/j.jns.2015.10.041. 26671114

[10] C. G. Bachmann , R. Rolke , U. Scheidt , et al., “Thermal Hypoaesthesia Differentiates Secondary Restless Legs Syndrome Associated With Small Fibre Neuropathy From Primary Restless Legs Syndrome,” Brain 133, no. Pt 3 (2010): 762–770, 10.1093/brain/awq026. 20194142

